# Distinct Requirements for HIV-1 Accessory Proteins during Cell Coculture and Cell-Free Infection

**DOI:** 10.3390/v11050390

**Published:** 2019-04-26

**Authors:** Anastasia Zotova, Anastasia Atemasova, Alexey Pichugin, Alexander Filatov, Dmitriy Mazurov

**Affiliations:** 1 Cell and Gene Technology Group, Institute of Gene Biology RAS, 34/5 Vavilova Street, 119334 Moscow, Russia; ashunaeva@gmail.com; 2 Faculty of Biology, Lomonosov Moscow State University, 1-12 Leninskie Gory, 119991 Moscow, Russia; justatemasova@gmail.com; 3 NRC Institute of Immunology FMBA of Russia, 24 Kashirskoe Shosse, 115472 Moscow, Russia; pichalvas@gmail.com (A.P.); avfilat@yandex.ru (A.F.)

**Keywords:** HIV-1, accessory proteins, restriction factors, cell-to-cell infection, BST2, Vpu, Nef, CRISPR-Cas9 knockout

## Abstract

The role of accessory proteins during cell-to-cell transmission of HIV-1 has not been explicitly defined. In part, this is related to difficulties in measuring virus replication in cell cocultures with high accuracy, as cells coexist at different stages of infection and separation of effector cells from target cells is complicated. In this study, we used replication-dependent reporter vectors to determine requirements for Vif, Vpu, Vpr, or Nef during one cycle of HIV-1 cell coculture and cell-free infection in lymphoid and nonlymphoid cells. Comparative analysis of HIV-1 replication in two cell systems showed that, irrespective of transmission way, accessory proteins were generally less required for virus replication in 293T/CD4/X4 cells than in Jurkat-to-Raji/CD4 cell cocultures. This is consistent with a well-established fact that lymphoid cells express a broad spectrum of restriction factors, while nonlymphoid cells are rather limited in this regard. Remarkably, Vpu deletion reduced the level of cell-free infection, but enhanced the level of cell coculture infection and increased the fraction of multiply infected cells. Nef deficiency did not influence or moderately reduced HIV-1 infection in nonlymphoid and lymphoid cell cocultures, respectively, but strongly affected cell-free infection. Knockout of BST2—a Vpu antagonizing restriction factor—in Jurkat producer cells abolished the enhanced replication of HIV-1 ΔVpu in cell coculture and prevented the formation of viral clusters on cell surface. Thus, BST2-tethered viral particles mediated cell coculture infection more efficiently and at a higher level of multiplicity than diffusely distributed virions. In conclusion, our results demonstrate that the mode of transmission may determine the degree of accessory protein requirements during HIV-1 infection.

## 1. Introduction

The human immunodeficiency virus type 1 (HIV-1), a causative agent of AIDS pandemic discovered more than thirty years ago, belongs to the family of complex retroviruses, whose genome, in addition to *gag-pol* genes, encodes regulatory and accessory proteins that lack any viral structural or enzymatic activity. It has been known for a long time that mutations in accessory genes decrease viral infectivity. This was a reason to call them viral infectivity proteins/factors. Later on, a number of host cellular proteins, called restriction factors have been discovered, as targets for viral accessory proteins. It turned out that many of them could confine the replication not only for HIV, but also for other viruses [1]. Although the number of discovered restriction factors targeting HIV is growing every year, some aspects of HIV restriction remain poorly understood. Today it is known that restriction factors affect virtually all stages of HIV replication cycle: capsid uncoating (TRIM5) [2,3,4], reverse transcription (APOBEC3 [5,6,7] and SAMHD1 [8,9,10]), nuclear import and integration (MxB) [11,12,13], translation (Schlafen 11) [14,15], budding (BST2/Tetherin) [16], and entry (SERINC5) [17,18]. HIV has evolved its own proteins Vif, Vpu, Vpx/Vpr, and Nef that efficiently counteract restriction factors by neutralizing them in a different and often sophisticated manner. One of these viral proteins, Vpu, was believed to make HIV-1 group M pandemic [19], unlike HIV-1 group O, N, or P.

The role of accessory proteins and restriction factors in HIV replication has been studied extensively upon infection with cell-free viruses. However, along with the classical transmission via cell-free viruses, HIV uses different cell–cell contacts, such as membrane nanotubes, filopodial bridges, and the virological synapse (VS), to transmit directly from an infected cell to a susceptible target cell (reviewed in [20]). This route of transmission considered to be a very efficient and important for HIV dissemination and pathogenesis both in vitro [21] and in vivo [22,23]. Cell-to-cell transmission has been difficult to quantify precisely because the previously infected cells and the newly infected cells are mixed together in the same culture. In addition, current methods utilizing replication-competent virus to measure cell-to-cell infection are limited when experiments require viral gene mutagenesis. Inferred by these limitations, the studies of restrictions factors at cell–cell mode of HIV replication remain incomplete, and, we believe, produce controversial results, whether, for instance, BST2/tetherin decreases cell-to-cell spread of HIV, as it does for cell-free viruses [24,25,26,27,28], or cell-to-cell transmission helps HIV to overcome cellular restriction [29,30].

Here, we generated four HIV-1 packaging vectors with single mutations in *vif*, *vpr*, *vpu*, or *nef* accessory gene. Using improved replication-dependent inLuc vector [31] we quantified the levels of replication for wild type (wt) and mutant HIV-1 at cell-free and cell coculture settings in lymphoid Jurkat-to-Raji/CD4 and nonlymphoid 293T/CD4/X4 cells. The replication-dependent vectors have been engineered to prevent a functional reporter protein expression in a transfected cell by reverting the reporter expression cassette relative to viral genome and interrupting reporter gene with an intron. However, once the reporter RNA is spliced out in a producer cell and packaged into VLPs, which then infect a target cell, the viral RNA is reverse transcribed and starts to produce a functional reporter protein [32]. Thus, these vectors are capable of measuring cell coculture infection of HIV-1, which represents a sum of cell-to-cell and cell-free infection, with no need to separate transfected and target cells, since no signal from a transfected cell is generated. In this study, we demonstrated that Vpu deletion enhanced HIV-1 cell coculture but not cell-free infection. Likewise, mutation in Nef more severely affected cell-free HIV-1 replication than replication of HIV-1 in cell cocultures. Negative effects of Vif or Vpr mutation on HIV-1 infectivity that we observed only in lymphoid cells were little dependent on mode of transmission. By cotransfecting two fluorescence reporter vectors inGFPt and inmCherry we also demonstrated that Vpu deletion enhanced the multiplicity of HIV-1 cell coculture infection. In knockout/restoration experiments, we confirmed that BST2 mediated the formation of Vpu(-) viral aggregates on producer T cells, which were responsible for the enhanced replication of Vpu(-) HIV-1 in cell cocultures. Our results demonstrate that in some cases the mode of transmission may determine the degree of accessory protein requirements during HIV-1 replication.

## 2. Materials and Methods

### 2.1. Cell Cultures and Reagents

The human embryonic kidney 293T cells were obtained through NIH AIDS Research and Reference Reagent Program. The human CD4 T cell line Jurkat E6 was purchased from ATCC. The human B cell line Raji/CD4 was kindly provided by Vineet N. Kewal Ramani (NCI at Frederick, MD, USA). All suspension cells were maintained in RPMI culture medium supplemented with 10% fetal bovine serum, 2 mM l-glutamine, and 40 µg/mL of gentamicin. To select for CD4 expression, 400 µg/mL of Geneticin (Gibco, Gaithersburg, MD, USA) was added to the culture of Raji/CD4 cells. HEK 293T cells were grown in DMEM culture medium containing 10% fetal bovine serum, 50 µM β-mercaptoethanol, 2 mM l-glutamine, and 40 µg/mL of gentamicin. Phorbol 12-myristate 13-acetate (PMA) was purchased from Sigma. The primary mouse monoclonal antibody (mAb) against human CD4 clone LT4 was from Sorbent (Moscow, Russia), anti-human CXCR4 mAb clone 12G5 was purchased from Santa Cruz Biotechnology (Dallas, TX, USA), anti-BST2 (CD317) mAb was from Biolegend (San Diego, CA, USA), anti-core HIV-1 antigen FITC-conjugated mAb clone KC57 was purchased from Beckman Coulter (Brea, CA, USA), and anti-HTLV-I p19 mAb clone 45/6.11.1. was obtained from ZeptoMetrix™ Corporation (Fisher Scientific, Waltham, MA, USA). The secondary goat anti-mouse Ab conjugated to Alexa488 Fluor were purchased from Invitrogen (Thermo Fisher Scientific). The polyclonal rabbit Abs against HIV-1 Vpu, Vpr, Vif, and Nef were obtained through NIH AIDS Research and Reference Reagent Program. The hybridoma clone 12G10 producing anti-alfa-tubulin mAb was obtained through the Developmental Studies Hybridoma Bank at the University of Iowa. The human recombinant interferon-alfa-2b (Altevir) was purchased from PharmaPark LLC (Moscow, Russia).

### 2.2. Plasmids

The mutations in accessory genes were generated in HIV-1 packaging vector pCMV-dR8.2 (Addgene, Watertown, MA, USA) which encodes for viral proteins from the NL4-3 strain of HIV-1. To mutate *vif* and *vpr* genes, the DNA fragment of 892 bp was cut out from pCMV-dR8.2 plasmid using *Afl*II restriction sites, blunted with Klenov polymerase and subcloned into *Sma*I-digested pKS Bluescript vector resulting in *Afl*II site reconstitution. The viral DNA fragment in a small plasmid was then mutagenized using Quick Change PCR Mutagenesis protocol with the following pairs of primers; 5-GGATTAACACATGAAAAAGATTAGTAAAACACCATATGTATATTTC-3 and 5-GTTTTACTAATCTTTTTCATGTGTTAATCCTCATCCTGTC-3 for *vif* mutation and 5-CAAGGGCCATAGAGGGAGCCATACAATGAATGG-3 and 5- GCTCCCTCTATGGCCCTTGGTCTTCTGGG-3 for *vpr* mutation. The mutation in *vpu* gene was generated in a similar way, except that the 1.4 kb fragment of viral genome flanked by *Bam*HI/*Sal*I restriction sites was subcloned into pKS Bluescript vector and PCR-amplified with the primers 5-GTAGCAATAGTAGCATAAGTAGTAGCAATAATAATAGCAATAGTTG-3 and 5-CTATTATTATTGCTACTACTTATGCTACTATTGCTACTATTATAGGTTG-3. All mutations have been verified by sequencing, and then the fragments of viral genome were transferred back into the HIV-1 packaging vector. To obtain *nef* mutation, pCMV-dR8.2 plasmid DNA was digested at a unique *Xho*I restriction site, which is located at 5′-terminus of *nef* gene. The DNA ends were blunted with Klenov polymerase and religated, which resulted in generation of a frame-shift mutation. The HIV-1 replication-dependent pUCHR-inLuc-mR, pUCHR-inGFPt-mR, and pUCHR-inmCherry-mR reporter vector improved by insertion of miR30 shRNA within the intron were described earlier [31]. The HIV-1 Env expression plasmid pIIINL4env [33] was obtained from Eric Freed (NCI at Frederick, MD, USA). The CD4 and CXCR4 coding sequences were PCR-amplified using respective primers and cDNA obtained from Jurkat cells, verified by sequencing, and cloned into lentiviral vector pUCHR at *Eco*RI/*Xma*I restriction sites. The guide-RNA (gRNA) expression vector pKS gRNA BB and the plasmid for the expression of nickase mutant D10A of Cas9 in a pcDNA 3.3 vector (Addgene #41815), were constructed earlier [34]. The pair of gRNAs for double nicking (DN) knockout of the human BST2 gene with the target sequences 5-TCTGCTGGGGATAGGAATTC-3 and 5-GCGCTTATCCCCGTCTTCCA-3 was selected using web-based resource http://tools.genome-engineering.org, and designed to clone into pKS gRNA BB vector via BbsI restriction site.

### 2.3. Transfections and Infections

The human 293T cells were transfected with Lipofectamine 2000 (Invitrogen, Carlsbad, CA, USA) according to the manufacturer’s instruction. The Jurkat cells were transfected with Neon electroporation system (Invitrogen) using three 10-ms pulses at 1350V. For VLP production, the Jurkat cells were transfected with Mirus TransIT^®^2020 transfection reagent in accordance to manufacturer’s protocol. The 293T/CD4/X4 cells permissive for NL4-3 HIV-1 were generated by sequential transduction of HEK 293T cells with pUCHR-CD4 and pUCHR-CXCR4 lentiviral constructs and FACS-sorting of antigen-positive cells. The HIV-1 one-step transfection/infection assay was set up by cotransfecting 293T/CD4/X4 cells in a 12-well plate with 0.6 µg of original or mutated pCMV-dR8.2 plasmid DNA, 0.15 µg of pIIINL4env vector DNA, and 0.9 µg of reporter plasmid pUCHR-inLuc-mR. Culture medium was replaced 6 h post-transfection. Samples were harvested and analyzed at 48 h post-transfection. Cells were resuspended (at this moment a small aliquot of sample was taken for lysis and total Gag measurement), pelleted, and lysed using the GLO lysis buffer (Promega, Madison, WI, USA). The levels of infection were estimated by measuring luciferase activity using Promega luciferase reagent and GloMax^®^-Multi Jr Single-Tube Multimode Reader (Promega). The supernatants were clarified through 0.45 µm pore size filters and used both for p24 quantitation and to set up a cell-free infection with new freshly plated 293T/CD4/X4 targets cells. The cell coculture infection assay in lymphoid cells was performed as described [31,32,35]. Briefly, 2 × 10^6^ Jurkat cells were cotransfected with 3 µg of pUCHR-inLuc-mR (or 3 µg of pUCHR-inGFPt-mR + 3 µg of pUCHR-inmCherry-mR), 2 µg of pCMV-dR8.2 plasmid DNA with or without mutation in an accessory gene, and 0.8 µg of pIIINL4env plasmid. After 24 h transfection, cells were resuspended (at this moment a small aliquot of sample was taken for lysis and total Gag measurement) and mixed with 10^6^ Raji/CD4 target cells in 5 ml of culture medium. At 72 h, cells were resuspended and analyzed by flow cytometry in case of transfection with fluorescence reporter plasmids, or lysed to measure luciferase activity, when the test was set up with inLuc vector. For cell-free infection assay, 10^7^ Jurkat cells in 10-cm dishes were cotransfected overnight with 6 µg of pUCHR-inLuc-mR, 4 µg of pCMV-dR8.2 plasmid DNA with or without mutations in accessory genes, and 1.6 µg of pIIINL4env plasmid. The next day, the culture medium was replaced, cells were incubated for 72 h, and pelleted. The supernatants were harvested and filtered. VLPs in the supernatants were concentrated by centrifugation at 100,000× *g* for 1 h at 4 °C, resuspended in 1 mL of culture medium, and added immediately to 10^6^ Raji/CD4 cells overnight. The small aliquots of concentrated VLPs were stored for a subsequent p24 ELISA test. Next day, infected cells were transferred to a 6-well plate in 5 mL of culture medium and incubated for 72 h before analysis. Sixteen hours prior harvesting, lymphoid cells, both in cell-free and cell coculture experiments, were stimulated with 20 nM of phorbol 12-myristate 13-acetate (PMA) to enhance luciferase expression from CMV promoter in infected cells.

### 2.4. Western Blot

The HEK 293T cells transfected with wt or mutant pCMV-dR8.2 plasmid DNA for 48 h were washed in PBS and extracted in 1 mL of ice-cold lysis buffer containing 1% Triton X-100 (Sigma, St. Louis, MO, USA), 150 mM NaCl, 10 mM Tris (pH 7.5), and a protein inhibitor cocktail (Complete Mini; Roche Applied Science, Basel, Switzerland). After 1 h of extraction at 4 °C, insoluble material was removed by centrifugation at 20,000× *g* for 15 min. The proteins in 1× SDS sample buffer containing reducing agent were resolved by 15%-PAGE and transferred onto 0.22-µm pore size PVDF membrane (Millipore, Billerica, MA, USA) by semidry blotting method using Mini Trans-Blot apparatus (Bio-Rad, Hercules, CA, USA). Membranes were blocked overnight at 4 °C with 5% (*w*/*v*) nonfat milk in PBS containing 0.1% Tween 20, probed with primary antibodies, washed with PBS-Tween and developed with horseradish peroxidase-conjugated secondary antibodies (Cell Signaling, Danvers, MA, USA). Blots were washed again, and immunoreactive bands were detected with Immobilon Western reagent (Millipore, Burlington, MA, USA) on a Molecular Imager ChemiDoc XRS (Bio-Rad).

### 2.5. Flow Cytometry and Cell Sorting

Cell surface staining was performed in PBS solution containing 0.1% sodium azide, 0.5% bovine serum albumin (BSA), and 5 µg/mL of primary mAb for 30 min on ice. Cells were washed twice with PBS, incubated with anti-mouse Alexa488-conjugated Ab diluted as indicated for primary Ab for another 30 min at +4 °C. After washing with PBS, the labeled cells were analyzed by flow cytometry. To generate polyclonal population of BST2 KO cells, Jurkat cells were electroporated with 3 µg of pcDNA 3.3 Cas9 D10A plasmid, 1 µg of pKS gRNA-BST2-1 plasmid, and 1 µg of pKS gRNA-BST2-2 plasmids designed to express gRNA for DN (double nicking) of *bst2* gene. Transfected cells were cultured and passaged for a week to develop KO phenotype, then stimulated with 10^4^ U/mL interferon-α2b overnight, and stained for surface BST2 as described above. The BST2-negative population was sorted in a single tube using FACSAria II Becton Dickinson Instrument. The sorted cells were grown, stained for BST2 again, and subjected to another round of sorting to achieve KO cells purity >98%. The similar sorting and Ab staining procedures were performed for isolating 293T cells stably transduced to express CD4 and CXCR4 antigens. Flow cytometry analysis was performed on Cytoflex S instrument (Beckman Coulter) using the 488-nm laser for excitation of Alexa488 and GFPt, and the 561-nm laser for excitation of mCherry. The collected data were processed with CytExpert software (Beckman Coulter) and presented using FlowJo (FlowJo LLC, Ashland, OR, USA).

### 2.6. Fluorescence Microscopy

For virion localization experiments, wt or BST2 KO Jurkat cells transfected overnight with a HIV-1 or HTLV-1 viral packaging vector were adhered to glass coverslips covered by poly-L-lysine (Sigma). Cells on coverslips were gently washed with PBS, fixed in 4% paraformaldehyde solution for 20 min. and permeabilized in PBS buffer containing 0.2% saponin (Sigma) and 0.5% BSA for 30 min. The primary HIV-1 or HTLV-1 anti-Gag mAbs were added directly to the permeabilizing solution to the final concentration of 1 µg/mL for 1 h. Cells were washed with permeabilizing solution twice and stained with the secondary anti-mouse Alexa488-conjugated Ab in the same buffer and at the same concentration. After washing with permeabilizing solution and PBS, coverslips were transferred onto microscopy slides and maintained in Dako Cytomation Fluorescent Mounting Medium. The fluorescence images were analyzed on Olympus IX-71 inverted epifluorescence microscope using excitation/emission filter units for FITC (470–495 nm/510–550 nm) and equipped with Z-axis-motorized objective revolver controlled by Olympus cellSens Dimension software via Olympus Ix2–UCB Microscope Controller. Twenty slices in Z-stack with 0.3 µm distance in-between were captured and deconvolved using cellSens Dimension and Autoquant X3 software, respectively. The Maximum Likelihood Estimation (MLE) Z-stack projections were created using Fiji image processing software. Virus cluster quantification was performed on a binary image of (MLE) Z-stack projections. Yen method was chosen to perform auto threshold calibration. The resulting binary image was processed with a Watershed option to separate tightly coupled clusters. An automatic small cluster counter process was performed on a watershed image to count a total number of clusters in image. Different cells from different slides were analyzed to obtain representative results.

### 2.7. ELISA

The levels of HIV-1 Gag in resuspended cell cultures and supernatants were measured using HIV-1 p24 ELISA Kit (Zeptometrix, Buffalo NY, USA) in accordance to the manufacturer’s instruction.

### 2.8. Statistical Analysis

*p*-values were calculated using Student’s two-tailed unpaired *t*-test, and significance was defined as indicated in figure’s legends. Data are shown as a mean ± standard deviation. All experiments were repeated at least three times.

## 3. Results

### 3.1. Generation and Characterization of HIV-1 with Mutations in Accessory Genes

**Figure 1:**
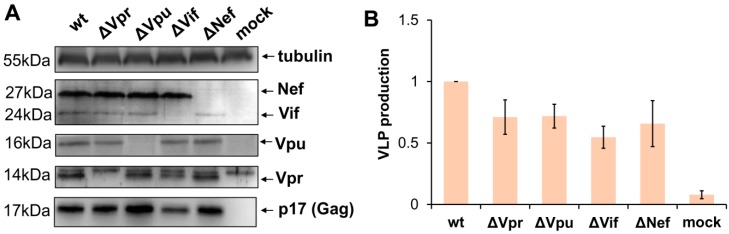
Characteristics of the generated accessory gene mutants of HIV-1. (**A**) Western blot analysis of HIV-1 accessory protein expression in 293T cells transiently transfected with the original (wt) pCMV-dR8.2 packaging plasmid or the plasmid containing mutation in one of the accessory genes (indicated as ∆) or mock plasmid. The blots were stained for viral proteins or tubulin as shown on the right. The molecular weights of stained proteins are indicated on the left. The blots with typical staining are presented. (**B**) The levels of VLP production by 293T cells transfected with wt or mutant HIV-1 packaging plasmid. The supernatants from transfected cells were harvested 48 h post-transfection, filtered, and analyzed using p24 ELISA Kit. The data obtained from the three independent experiments were calculated relative to wt control, and presented as the average values with the standard deviations.

To evaluate impact of viral accessory proteins on HIV-1 replication, we generated four HIV-1 packaging plasmids pCMV-dR8.2 with inactivating mutation in one of the accessory genes. Nef was mutated via cleavage of the viral genome with XhoI restriction enzyme followed by blunting with Klenov fragment and religation. The mutations generating premature stop codons in Vpu, Vif, and Vpr genes were obtained by Quick Change PCR mutagenesis as outlined in Methods section. As the 3′-terminal part of Vif overlaps the 5′-end of Vpr in HIV-1 viral genome, the Vpr mutation was designed so that it generates an early premature stop codon in a Vpr-reading frame but keeps the amino acid coding of Vif unchanged. After PCR mutagenesis, all fragments of HIV-1 genome were analyzed by DNA sequencing to ensure that there were no unwanted mutations, and then cloned back to the packaging vector. In order to verify whether the designed mutations abolish the expression of the appropriate accessory proteins and retain the production of virus like particles (VLPs), 293T cells were transfected with the wild type or mutant pCMV-dR8.2 vector or mock plasmid. In 48 h, cells were harvested, lysed, and analyzed by WB with antibodies (Abs) against HIV-1 accessory proteins, p17 matrix protein, or anti-tubulin mAb for protein load control (Figure 1A). The supernatants from transfected 293T cells were clarified through the 0.45-µm pore size filters, and the levels of VLPs production were quantified using p24 ELISA Kit (Figure 1B). As demonstrated in Figure 1, all generated mutations were specific, and inactivated expression of only one of four targeted HIV-1 accessory genes. The levels of Gag expressed in cells and released to the medium after transfection with mutant constructs were slightly-to-moderately altered when compared to Gag produced from wt packaging vector. Hence, for accurate evaluation of wt and mutant HIV-1 replication in subsequent experiments, we used normalization of infectivity data to the levels of Gag as specified below.

### 3.2. The Requirements for Accessory Gene Expression during HIV-1 Replication in Nonlymphoid Cells

**Figure 2:**
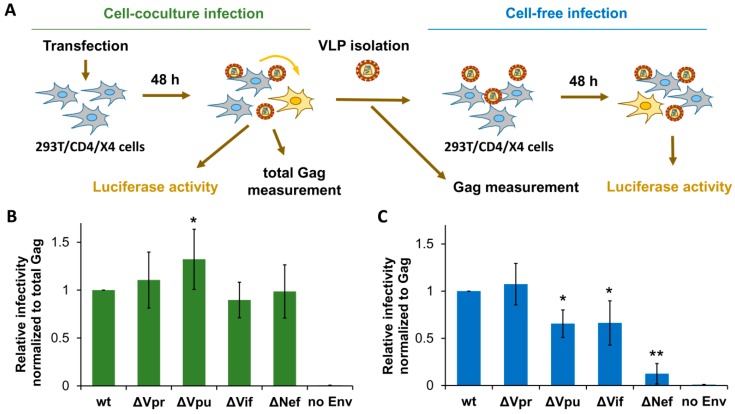
Effects of accessory gene inactivation on HIV-1 replication in HEK 293T/CD4/X4 cells. (**A**) Schematic representation of experiments designed to quantify and normalize HIV-1 infection in 293T/CD4/X4 cells. The levels of infection estimated by luciferase activity were normalized to the levels of Gag quantified by ELISA. The normalized level of infectivity obtained for wt HIV-1 was set at 1.0, and the infectivity levels for mutants were recalculated relative to that value. The levels of HIV-1 relative infectivity measured in 293T/CD4/X4 cell culture and cell-free infection tests are shown in (**B**,**C**), respectively. All data are representative of at least three independent experiments and shown as average values ± standard deviations. *, **, statistically different from the control (wt) by Student’s *t*-test at *p* < 0.05 and *p* < 0.01, respectively.

First, we estimated the replication potencies for generated HIV-1 mutants in HEK 293T cells at cell-free and cell coculture experimental settings. To simplify comparative analysis of virus replication in these cells and in lymphoid cells (described below), we generated HIV-1 permissive 293T/CD4/X4 cells and used identical vectors to measure HIV-1 replication in all subsequent tests. The 293T/CD4/X4 cell coculture infection was initiated by cotransfecting cells with wt or mutant HIV-1 packaging vector, pUCHR-inLuc-mR reporter vector [31], and expression plasmid encoding for NL4-3-derived Env. In this test, called the one-step transfection/infection assay [31], the transfected cells produce VLPs which then infect neighboring cells. To rule out the possibility that high level of transfection reduces availability of target cells for infection, we mixed transfected and nontransfected 293T/CD4/X4 cells at 1:1 ratio and measured infection 48 h thereafter. Since relative levels of infection measured by two types of tests were not significantly different, we further adhered to one-step assay. At 48 h post-transfection, cells were lysed, and the levels of cell-to-cell infection were quantified by measuring luciferase activity, while the supernatants were harvested, filtered through 0.45-µm pore size filters, and used to infect freshly cultured 293T/CD4/X4 target cells for another 48 h (cell-free infection) (Figure 2A). Given that both production and release of VLPs can be altered by mutations in HIV-1 accessory genes, we normalized cell coculture infection data to the levels of total Gag measured in aliquots of resuspended cells at the end of the assay. Cell-free infection was adjusted to the input of p24 used to initiate infection. The levels of normalized infectivity obtained for mutant HIV-1 were then compared to that detected for wt HIV-1, the value of which was set at 1.0. As demonstrated in Figure 2B,C, Vpr and Vif were either not or poorly required for HIV-1 replication in 293T cells. In contrast, Vpu deficiency increased HIV-1 replication 1.3-fold in cell cocultures, but decreased it in the cell-free setting. Interestingly, deletion of Nef was dispensable for HIV-1 replication in cell cocultures, but severely reduced infectivity of purified VLPs. Thus, except Nef, which was strongly required for cell-free infection, the requirements for accessory proteins during HIV-1 replication in 293T cells were low.

### 3.3. Effects of Accessory Gene Mutations on HIV-1 Cell Coculture and Cell-Free Infection in Lymphoid Cells

**Figure 3:**
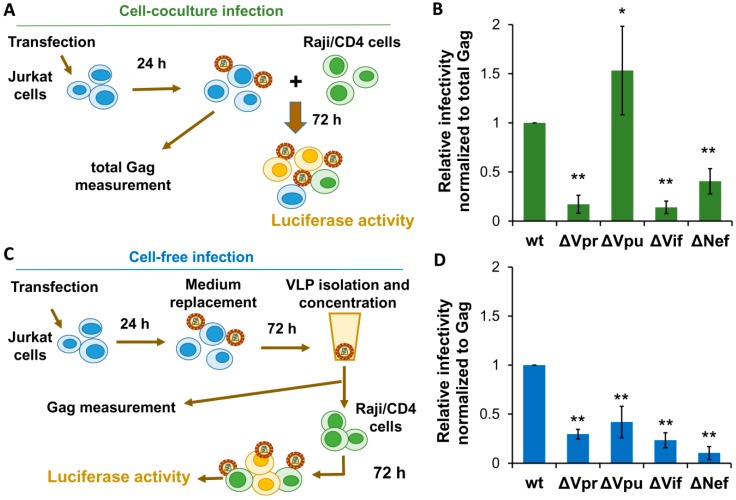
The requirements for HIV-1 accessory proteins during HIV-1 replication in lymphoid cells. (**A**) Schematic experimental design for evaluation HIV-1 replication in Jurkat-Raji/CD4 cell cocultures. (**B**) The results of HIV-1 relative infectivities measured in Jurkat-to-Raji/CD4 cell cocultures. (**C**) Scheme of experimental set up for detecting infectivity of HIV-1 VLPs derived from Jurkat cells and applied to Raji/CD4 target cells. (**D**) The levels of infectivity of VLPs purified from Jurkat cells and use to infect Raji/CD4 cells. The levels of replication were calculated as described for Figure 2 and as specified in the Results section. The data shown as average values ± standard deviations are representative of at least three independent experimental repeats. *, **, statistically different from the control (wt) at *p* < 0.05 and *p* < 0.01, respectively.

To evaluate HIV-1 replication in cocultures of human lymphoid cells, we used Jurkat-to-Raji/CD4 infectivity assay described earlier [31,32,35]. Briefly, 2 × 10^6^ Jurkat producer cells were cotransfected with wt or mutant HIV-1 packaging vector, inLuc-mR reporter plasmid, and NL4-3 Env expression vector. Next day, transfected cells were resuspended (at this stage a small aliquot of suspension was saved for a total Gag quantification) and mixed to the 10^6^ target Raji/CD4. Taking into account that, on average, 40–50% of Jurkat cells were transfected, the ratio of producer cells to target cells was close to 1:1. Three days later, cells were lysed, and luciferase activity was measured (Figure 3A). The relative levels of infectivity were calculated as outlined above for cell culture infection in 293T/CD4/X4 cells. As shown in Figure 3B, Vpu inactivation resulted in a 1.5-fold enhancement of HIV-1 infectivity in coculture of Jurkat producer cells with Raji/CD4 target cells (Figure 3B), which was stronger than the similar effect observed in 293T/CD4/X4 cell culture (Figure 2B). In contrast, mutations in Vpr, Vif, or Nef, which did not influence substantially HIV-1 replication in 293T/CD4/X4 cells, decreased the levels of HIV-1 replication in Jurkat-Raji/CD4 cocultures 5.9-, 7.7-, and 2.6-fold, respectively.

The cell-free infection from Jurkat cells (it was important to keep lymphoid origin of producer cells) to Raji/CD4 target cells was set up by transfecting Jurkat cells in a large scale to generate VLPs in amount sufficient for infectivity detection. Briefly, 10^7^ cells in 10 ml of culture medium were cotransfected with 4 µg of wt or mutant packaging vector, 6 µg of inLuc-mR reporter vector, and 2 µg of NL4-3 Env expression plasmid. In 24 h, the culture medium was replaced, and cells were incubated for 3 days. VLPs in cell culture supernatants were filtered, concentrated by centrifugation, resuspended in 1 ml of growth medium, and added immediately to 10^6^ Raji/CD4 cells. The small aliquots of concentrated VLPs were saved for Gag measurement. One day later, infected Raji/CD4 cells were transferred to the 5 mL of culture medium, and incubated for another 72 h (Figure 3C). Data were calculated and presented as described above for 293T/CD4/X4 cells. As shown in Figure 3D, all accessory proteins were strongly required for HIV-1 cell-free infection in lymphoid cells (the degrees of inhibition varied from 2.2-fold for Vpu to 9.7-fold for Nef). The effects of Vpr and Vif deletion on HIV-1 infectivity in lymphoid cells were comparable and little dependent on mode of transmission. In contrast, Vpu deficiency decreased cell-free infection, but enhanced infection in cell cocultures. Likewise, Nef was four-fold less required for HIV-1 infectivity in cell cocultures than for infectivity of purified VLPs.

In conclusion, both Vpr and Vif were strongly required for HIV-1 replication in lymphoid cells regardless transmission route, whereas Vpu and Nef depletion exerted distinct effects on HIV-1 replication depending on cell-free or cell-to-cell mode of transmission.

### 3.4. Vpu Deletion Increases Multiplicity of HIV-1 Infection in Lymphoid Cell Coculture

**Figure 4:**
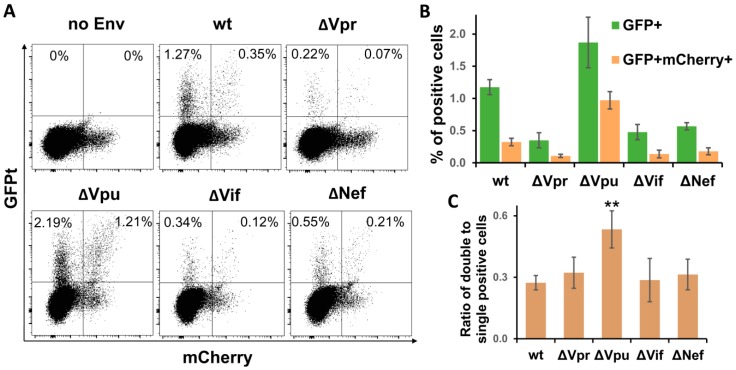
Quantification of cells infected by wt or accessory gene mutant HIV-1 in Jurkat-Raji/CD4 cell coculture using inGFPt and inmCherry vectors. (**A**) Typical flow cytometry DotPlot graphs showing distribution of single- and double-infected cells at day 3 after cell mixture. Quantitative analysis of single infected (GFP^+^) and double infected (GFP^+^ mCherry^+^) cells (**B**) and their ratios as a measure of MOI (**C**). Data are presented as average values ± Std dev from three independent experiments. All values obtained for indicated mutants of HIV-1 in B are statistically different from wt control by Student’s *t*-test at *p* < 0.01. **, statistically different from the control (wt) at *p* < 0.01.

Earlier, we have explored a pair of improved intron-regulated fluorescence-based reporter vectors to demonstrate that HIV-1 cell-to-cell transmission enhances the level of multiplicity of infection (MOI), and that HIV Env is responsible for this enhancement [31]. Unlike luciferase measurement, fluorescent reporters are capable of detecting individual infected cells, and, in case of using two different fluorescent proteins, cells infected by two or more VLPs, i.e., multiply infected cells, can be quantified. We took an advantage of using these reporters and evaluated effects of HIV-1 accessory gene deletions on MOI. Of all viral replication tests described above, the sensitivity of fluorescent reporters was sufficient to measure HIV-1 infection only in Jurkat-Raji/CD4 cell coculture. The assay was performed essentially as described in previous paragraph, but instead of inLuc reporter, Jurkat cells were cotransfected with 3 µg of inGFPt and 3 µg of inmCherry plasmids along with packaging and Env expressing vectors. After three days of coculture, the numbers of infected cells were evaluated by flow cytometry using two fluorescence parameters. The typical DotPlots recorded for wt and mutant HIV-1 are shown in Figure 4A. Due to high background fluorescence of cells in mCherry channel (see no Env control), we quantified percentages of GFP^+^ mCherry^+^ and GFP^+^ cells only, as well as ratios between them. As illustrated in Figure 4B, the levels of infectivity measured for HIV-1 accessory gene mutants using fluorescent reporter proteins corresponded well to the results obtained with luciferase (Figure 3B), i.e., deletion of Vpu enhanced the HIV-1 infectivity, while inactivation of Vpr, Vif, or Nef reduced it (normalization of RLU to Gag only slightly corrected infectivity). Thus, the effects of accessory gene mutation on HIV-1 infectivity were reproduced with fluorescent proteins.

To estimate MOI, we calculated the proportion of double-positive cells relative to single-positive cells (Figure 3C). This parameter evaluated for ∆Vpr, ∆Vif, and ∆Nef HIV-1 was not significantly different from wt control, though it can be expected that reduced overall infectivity of these mutants will result in decreased MOI as well. By contrast, Vpu deletion increased the fraction of double-positive cells almost twofold in comparison to wt HIV-1. Thus, using a pair of intron-containing fluorescent reporter vectors, we demonstrated that mutation in Vpu enhanced both the total level and the multiplicity of HIV-1 infection.

### 3.5. BST2 Knockout Abolishes Enhanced Replication of HIV-1 ΔVpu in Lymphoid Cell Coculture

**Figure 5:**
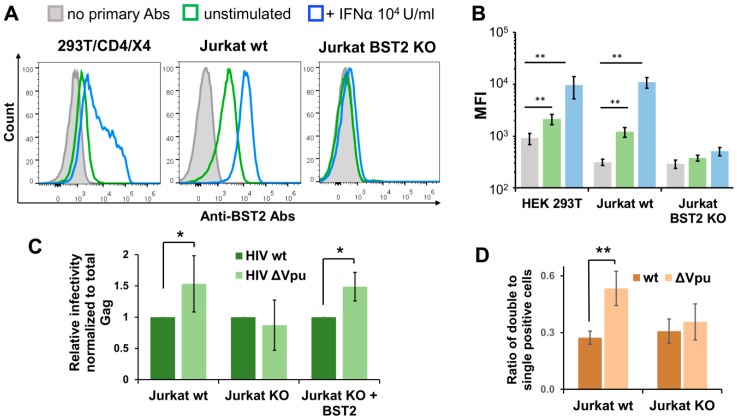
BST2 knockout and replication of Vpu-deficient HIV-1. (**A**) Flow cytometry analysis of spontaneous and INF-α2b-induced BST2 expression on surfaces of indicated cells. The data are shown as the representative overlaid FACS histograms (A) and as the levels of mean fluorescence intensity (MFI) (**B**). The color codes in B match those in A. (**C**) The levels of wt and ∆Vpu HIV-1 replication in Jurkat-Raji/CD4 cell cocultures were comparably examined using parental Jurkat cells (left two bars), BST2 KO Jurkat cells (middle two bars), and BST2 KO Jurkat cells transiently cotransfected with BST2 expression plasmid (right two bars). The infectivity data were normalized to the levels of total Gag and the values obtained for HIV-1 ∆Vpu were calculated relative to the respective wt HIV-1 controls. (**D**) The levels of MOI detected with wt or KO Jurkat cells and calculated as described for Figure 4C. The results in **B**–**D** are representative of at least three independent experiments and shown as averages with standard deviations. *, **, data are statistically different at *p* < 0.05 and *p* < 0.01, respectively.

Since the HIV-1 Vpu protein antagonizes the restriction factor BST2 (CD317/tetherin), we sought to investigate whether the BST2 mediates enhanced replication of HIV-1 ΔVpu. First, we examined the levels of spontaneous and interferon-α (IFNα)-induced BST2 expression on surfaces of 293T/CD4/X4 and Jurkat cells. In agreement with previous reports, untreated and IFNα-treated Jurkat cells expressed more BST2 than 293T/CD4/X4 cells (Figure 5A, two left histograms). This can partly explain the slightly higher effect of Vpu deletion on HIV-1 infectivity in Jurkat-Raji/CD4 coculture than in 293T/CD4/X4 cell culture. Next, we generated Jurkat cells with BST2 knockout (KO) using CRISPR/Cas9 technique. To minimize off-target effects and avoid potential biases in retroviral replication related to clonal isolation of gene-edited cells that we reported earlier [36,37], we combined double nicking (DN) approach [38] with a polyclonal isolation of BST2-negative cells after staining with respective antibody and FACS-sorting. As a result, the sorted population of Jurkat cells carried out BST2 null phenotype and did not upregulate BST2 in response to IFNα treatment (Figure 5A, right hand histogram, and B). The infection tests performed with inLuc reporter as described for Figure 3B demonstrated that there was no difference between replication of wt and Vpu-negative HIV-1 in coculture of transfected Jurkat BST2 KO cells with Raji/CD4 cells (Figure 5C, two middle bars). On the contrary, the replication of HIV-1 ΔVpu in parental Jurkat-to-Raji/CD4 cell coculture was 1.5-fold higher than the replication of wt HIV-1 (Figure 5C, two left bars). When a BST2 expression plasmid was cotransfected with the viral vectors into Jurkat KO cells, the effect of Vpu deletion on replication of HIV-1 in coculture with Raji/CD4 cells was completely restored (Figure 5C, two right bars). To estimate impact of BST2 KO on MOI, we set-up an infection assay with inGFPt and inmCherry vectors and calculated the relative numbers of double positive cells as outlined above for Figure 4C. In contrast to wt Jurkat cells, where Vpu deletion increased MOI (Figure 5D, two left bars), BST2 KO abolished the difference between MOI levels detected for wt and ΔVpu viruses (Figure 5D, two right bars). Altogether, these data demonstrate that BST2 is predominantly, if not solely, responsible for enhancement in overall infectivity and MOI of HIV-1 ΔVpu in lymphoid cell coculture.

### 3.6. BST2 Induces Clusterization of HIV-1 ΔVpu and HTLV-1 Viral Particles on the Surface of T Cells

**Figure 6:**
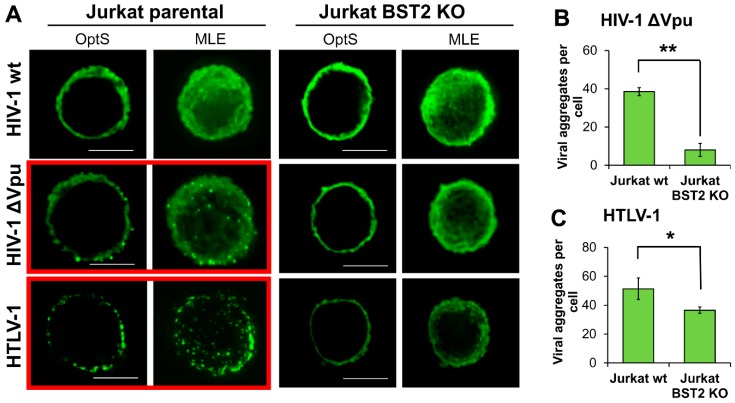
The patterns of HIV-1 and HTLV-1 viral particle expression in BST2-positive and negative T cell lines. (**A**) Jurkat parental or KO cells were transfected with wt or Vpu(-) HIV-1 or HTLV-1 packaging plasmid and followed fixation/permeabilization stained with the respective anti-Gag Ab. Cells were analyzed using fluorescence deconvolution microscope, and presented as a 0.3-µm optical slices through the middle plane of cells (OptS) or as Z-stack one plane projections obtained by Maximum Likelihood Estimation (MLE) algorithm (scale bar, 5 μm). At least 4-6 cells per sample were analyzed to demonstrate representative cells. The levels of clusterization for HIV-1 ∆Vpu (**B**) and HTLV-1 (**C**) were quantified using ImageJ Threshold, Watershed, and Analyze particles software options. A size limit for clusters was set to calculate numbers of clusters per cell. The data obtained from several cells and from different slides were averaged and presented. * and **, the differences between wild type and KO cells are statistically significant at *p* < 0.05 and *p* < 0.01, respectively.

It is reasonable to assume that BST2, which has a transmembrane domain at the N-terminus and a glycosylphosphatidylinositol (GPI) anchor at the C-terminus, can physically tether viruses to membrane as well as to each other and induce a formation of viral assemblies on T cells. These structures can be more efficient than diffusely expressed viruses in mediating viral spread upon direct contact of an infected cell with a target cell. To visualize viral particles on the surface of T cells, we transfected parental and BST2 KO Jurkat cells with pCMV-dR8.2 packaging vector with or without mutation in *vpu* gene, or with pCMV-HT1 packaging vector encoding wt HTLV-1 (Human T cell leukemia virus 1) genome. HTLV-1 is another human pathogenic retrovirus, whose spread is strikingly dependent on cell–cell transmission. It was included in the test, because, first, unlike HIV-1, it has no virally encoded factors that antagonize BST2, and second, the virus assembles into biofilm-like structures that are considered to be more infectious than cell-free virions [39]. The following day after transfection, cells were adhered to coverslips, fixed, permeabilized, and stained for HIV-1 or HTLV-1 Gag protein, respectively. Samples were analyzed using fluorescence deconvolution microscope, and the generated deconvolved Z-stack images of cells were shown as a 0.3-µm optical section through the middle plane of a cell (OptS) or as a maximum likelihood estimation projection (MLE), which represents an additional way to see virus distribution. As illustrated in Figure 6A, the viral particles were assembled into multiple aggregates, which were clearly visible on the surface of T cells when wt HTLV-1 or Vpu(-) HIV-1 were expressed in parental Jurkat cells (highlighted with red boxes). These results were expected, because under these circumstances BST2 can restrict HIV-1 release. Strikingly, on the surface of BST2 KO cells both HTLV-1 and HIV-1 ΔVpu viral particles were distributed diffusely. The described differences were confirmed statistically by setting threshold and size parameters for aggregates and calculating their numbers per cell using ImageJ software (Figure 6B,C, see section Methods for details). Thus, we concluded that BST2 is largely responsible for the formation of both HIV-1 and HTLV-1 viral aggregates at the plasma membrane of T cells. These viral assemblies mediated more efficient HIV-1 cell coculture infection than diffusely localized particles as was shown using replication-dependent reporter vectors (Figure 5C,D).

## 4. Discussion

It has been more than ten years since the first HIV restriction factors were discovered. The role and mechanisms of restriction, which have been well defined for a classical cell-free infection, leave an open window for debates when a restriction (for example, BST2-mediated) tackles cell-to-cell viral transmission [40,41]. In this study, using one cycle replication assay with the intron-regulated reporter vector, we demonstrated that HIV-1 accessory proteins are differently required for HIV-1 replication during cell coculture and classical cell-free infection. Of four HIV-1 accessory proteins, Vpu and Nef displayed the most drastic transmission-dependent effects on HIV-1 replication.

Currently, the restriction factors targeted by HIV-1 accessory proteins (except Vpr) are well established. The multispanning transmembrane proteins Serinc5 and to lesser extend Serinc3 inhibit HIV-1 fusion and uncoating, whereas Nef prevents Serinc incorporation into viral particles [17,42]. Recent studies suggest that Serinc5 is involved in HIV-1 Env conformational changes, which regulate fusion process and determine virion sensitivity to neutralizing antibodies [18]. Consistent with this, it has been shown earlier that the replication of VSVG-pseudotyped HIV-1 was not sensitive to Nef deletion [43]. We also tested the replication of VSVG-pseudotyped HIV-1 in 293T cells at different settings and found no significant effects of mutations in accessory genes, including Nef on viral infectivity (Zotova & Mazurov, unpublished data). Strikingly, HIV-1 with its own Env replicated poorly without Nef in cell-free infection tests (Figure 2C and Figure 3D), however, its infectivity was not affected by Nef in cocultures of 293T/CD4/X4 cells (Figure 2B) and was less dependent on Nef in Jurkat-Raji/CD4 cocultures than in cell-free test (compare Figure 3B,D). Given that VSVG exploits endocytic pathway of viral entry, we can draw some parallels between cell-to-cell transmission and endocytic pathway of HIV-1 entry, which can be resistant to Serinc restriction and, therefore, less dependent on Nef. Indeed, Dale et al. demonstrated that VS induces endocytosis of HIV-1 viral particles by target cells followed by fusion of viruses with endocytic membrane [44]. Such a scenario may benefit HIV-1 entry, if Serinc5, for instance, is sensitive to low pH, but this should be tested and proven experimentally. In addition to Serinc antagonism, Nef downregulates CD4 expression and greatly enhances infectivity of released virions [45]. As all producer cells in our experiments were CD4-positive, this mechanism may also contribute to the decrease in HIV ΔNef infectivity that we observed.

Another important restriction factor, whose role in HIV-1 cell-to-cell transmission has been debated, is BST2 (CD317, tetherin), an IFN-inducible type II transmembrane protein with a GPI-anchor. It acts in a producer cell and tethers nascent virions to the plasma membrane [46,47], which may then undergo endocytosis and degradation [16]. HIV-1 small membrane protein Vpu antagonizes BST2 through downregulation of its surface expression [48], ubiquitin-dependent proteasomal degradation [49,50,51] and autophagy [52]. Consistent with the early studies [53], we demonstrated that in the absence of Vpu HIV-1 viral particles form aggregates on the surface of T cells (Figure 6). The concurrent infection tests showed that the deletion of Vpu enhanced HIV-1 replication 1.5-fold in Jurkat-Raji/CD4 cell cocultures (Figure 3B), but decreased it more than twice when VLPs purified from Jurkat cells were used to infect Raji/CD4 (Figure 3D). Moreover, the deletion of Vpu increased the fraction of multiply HIV-1-infected cells in Jurkat-Raji/CD4 cell coculture (Figure 4). Although it has been reported that the restriction of cell-free infection quite well correlates with the level of BST2 expression, this was not observed often with cell-to-cell infection. In agreement with Clare Jolly report [29], we did not see enhancement in HIV-1 ΔVpu cell coculture infection after pretreatment of Jurkat cells with IFNα (Zotova & Mazurov, unpublished data), which upregulated BST2 and could induce an antiviral cellular response as well. Conversely, even low level of BST2 expression detected on our 293T/CD4/X4 cells can be responsible for mediating moderate enhancement in replication of HIV-1 with deleted Vpu (Figure 2B). The recent study demonstrated that artificial activation of BST2 promoter with dCas9 transcriptional complex inhibited replication of both wt and Vpu(-) HIV-1 [54]; authors concluded that dose-dependent interplay between Vpu and BST2 is important. Nevertheless, the clear-cut results that we obtained using BST2 null cells (Figure 5) confirmed the role of BST2 in the enhancement of cell-to-cell infection. Interestingly, we noticed that effects of Vpu deletion varied depending on cell type and Env. For example, in HeLa-CD4 cells expressing high level of BST2, Vpu deletion did not enhance HIV-1 infection in one-step assay (unpublished data). On the other hand, pseudotyping HIV-1 with VSVG makes the virus replication in 293T cells completely independent on accessory proteins. It should be kept in mind that Vpu not only antagonizes BST2, but also influences virion infectivity by downregulating CD4 expression on the surfaces of effector cells [45]. However, when we measured infection from 293T cells to 293T/CD4/X4 cells, the effect of Vpu deletion on HIV-1 infectivity remained the same (Zotova & Mazurov, unpublished data). This suggests that the engagement of HIV-1 Env on a producer cell with CD4 receptor on a target cell and establishing VS can be important for mediating enhanced replication of HIV-1 ΔVpu in cell coculture.

The human cytidine deaminase APOBEC3G (A3G) is another IFN-α-inducible restriction factor, which incorporates into budding viral particles and inhibits HIV-1 replication in target cells by hypermutating viral DNA during reverse transcription [5,6]. HIV-1 protein Vif mediates A3G binding to E3 ubiquitin ligase complex and recruits CBF-β to initiate A3G proteasomal degradation [55]. Primary T cells, macrophages and T cell lines such as CEM and H9 express high level of A3G and are nonpermissive for HIV-1 ∆Vif replication [56], while A3G expression in Jurkat cells is moderate [57]. A comparative analysis of transcriptome (http://www.transcriptonet.ca/mRNASpec.aspx) suggests that A3G in Jurkat cells is expressed 3.5-fold higher than in 293T cells. Hence, the level of A3G expression may explain why the infectivity of HIV-1 in lymphoid cells was dependent on Vif stronger than HIV-1 replication in 293T/CD4/X4 cells (compare results in Figure 2 and Figure 3). The cellular protein targeted by Vpr is unknown, though some evidences suggest that TRIM11 can be indirectly involved into Vpr antagonism [58]. We demonstrated that Vpr was not required for HIV-1 replication in 293T/CD4/X4 cells, but was substantially needed for virus replication in lymphoid cells both in coculture and cell-free settings. The higher level of TRIM11 expression in 293T cells than in lymphoid cells (https://www.proteinatlas.org/ENSG00000154370-TRIM11/cell) does not explain the observed differences and indicates that other unknown cellular factor/s can be involved in the sensitivity of HIV-1 replication to Vpr deletion.

In conclusion, our study refines the significance of cell-to-cell transmission for replication of HIV-1, which is defective in one of the accessory genes. We demonstrated that coculture of viral effector cells with target cells alleviated the replication of Nef-deficient HIV-1 and enhanced the replication of Vpu-deficient HIV-1 in comparison to wt virus. Notably, the deletion of Vpu increased the fraction of multiply infected cells as well. This can potentially increase the risk of HIV-1 recombination and generation of new resistant forms. Thus, understanding relationship between restriction and transmission can help develop more effective strategies to combat HIV.
